# Knowledge structure and research hotspots on digital scanning for implant-supported complete-arch prosthesis: A bibliometric analysis

**DOI:** 10.1016/j.heliyon.2024.e36782

**Published:** 2024-08-23

**Authors:** Yutong Gao, Mingyu Zhao, Shici Xia, Yue Sa

**Affiliations:** a Department of Prosthodontics, State Key Laboratory of Oral & Maxillofacial Reconstruction and Regeneration, Key Laboratory of Oral Biomedicine Ministry of Education, Hubei Key Laboratory of Stomatology, School & Hospital of Stomatology, Wuhan University, Wuhan, PR China; b Department of Prosthodontics, The State Key Laboratory Breeding Base of Basic Science of Stomatology (Hubei-MOST) & Key Laboratory of Oral Biomedicine Ministry of Education, School & Hospital of Stomatology, Wuhan University, Wuhan, PR China

**Keywords:** Digital scanning, Implant-supported complete-arch prosthesis, Bibliometric

## Abstract

**Background:**

Digital scanning is increasingly widely used for implant-supported complete-arch prosthese. However, a quantitative literature analysis is lacking for this field. This study aims to conduct a bibliometric analysis to summarize the knowledge structure and research hotspots of digital scanning for implant-supported complete-arch prosthesis.

**Materials and methods:**

Relevant articles and reviews, published between 1994 and 2023, were obtained from the Web of Science Core Collection (WoSCC). Indicators such as publication count, annual growth, citation count, co-citation count, impact factor, Journal citation reports (JCR) division, H-index are used to assess the contribution of countries, journals, authors or the quality of articles. Visual maps, cluster analysis and keyword cloud are used to evaluate the cooperation pattern and topic trends.

**Results:**

580 eligible publications, including 555 articles and 25 reviews, were analyzed. The United States is the leading country in this area, received the most citations. The Journal of Prosthetic Dentistry is the scientific journal with the highest impact. The analysis of keywords and ongoing trials shows that the accuracy of relevant techniques is a current hot topic in this field.

**Conclusion:**

In recent years, digital scanning technique for implant-supported complete-arch prosthesis has made rapid progress. By reviewing the published literature, we found the United States is the global leader in the field of digital scanning for complete-arch implant prosthesis. Accuracy is the core word in this field, more scientific evidence is needed to support the clinical application of digital scanning in this field.

## 1 Introduction

The fabrication of implant-supported complete-arch prosthesis is considered a challenging process in implant dentistry [1]. The conventional fabrication method starts with a physical impression, requiring a variety of intricate manual manufacturing steps, materials, equipment as well as experience and skills of the operator [2]. Over the past few decades, dental practice has been transformed by the introduction of digital scanning techniques. The advantages of digital scanning have been demonstrated in many studies, including reducing patient discomfort, saving time, eliminating the risk of deformation of impression materials, as compared to conventional impression [[3], [4], [5], [6], [7], [8], [9], [10], [11], [12], [13], [14], [15], [16], [17], [18]]. However, the application of digital scanning for implant-supported complete-arch prosthesis still faces some challenges.

Accurately acquiring the implant positions is crucial for the fit and long-term success of implant-supported prosthesis, as insufficient accuracy may lead to potential mechanical and biological complications [[3], [4], [5], [6]]. For edentulous arch, the conventional open-tray splinted impression remains the preferred choice clinically based on its high accuracy, which will be transferred to 3D digital model with an extraoral laboratory scanner [14,[19], [20], [21]]. However, there is a risk of impression deformation and triggering the gag reflex caused by the use of impression materials [22,23]. Currently, intraoral scanning (IOS) is a common solution for directly capturing the intraoral morphology data of the soft tissue and scan bodies [24,25]. Despite the lack of anatomic indexes of the edentulous arch creates challenges for IOS, recent studies have demonstrated that IOS achieves clinically acceptable accuracy when applied to edentulous implant arches [[26], [27], [28], [29], [30], [31], [32], [33]]. Furthermore, photogrammetry is also available for accurately capturing the 3D implant positions, which is commonly used in conjunction with IOS. By integrating the accurate implant position data acquired through photogrammetry and the mucosal morphology data acquired through IOS, a complete-arch digital implant scan can be achieved [34]. Digital scanning based on IOS and photogrammetry might provide a clinically acceptable alternative to conventional complete-arch implant impressions, which is currently a hot topic in oral clinical practice. Therefore, it is necessary to understand its overall knowledge structure and clinical application prospects.

To bridge the knowledge gap and further advance the field, a comprehensive report on knowledge structure and research hotspots of digital scanning for implant-supported complete-arch prosthesis is urgently needed. Currently, many scientific methods are employed to systematically comprehend the present condition of an academic discipline. Among these methods, bibliometric analysis utilizing mathematical and statistical approaches has emerged as a prevalent tool for gaining insights into the overall knowledge structure and research priorities within an academic domain [[35], [36], [37], [38]]. However, there have been no comprehensive analysis of global research trends of this field. This study aimed to use bibliometric methods to describe the current status of digital scanning for implant-supported complete-arch prosthesis, assess research prospects, and predict new trends in the development of this field.

## 2 Materials and methods

### 2.1 Study design

A descriptive and observational scientometric study was conducted using the Web of Science Core Collection (WoSCC) Citation Index Expanded (SCI-E) databases, which is a comprehensive database for scientific research [39]. The information retrieval process was performed on December 24, 2023 and the search covered the period of 1994–2023. The search strategy was formulated using the identified common keywords about digital scanning for implant-supported complete-arch prosthesis. Relevant publications were searched and downloaded for bibliometric analysis.

### 2.2 Search strategy

Only articles or reviews in English published between January 1, 1994, and December 24, 2023, were selected. Other types of publications were excluded, including meeting abstract, proceeding paper, book chapters, letter, editorial material, early access, correction and news item. According to the related literature, we identified the common keywords about digital scanning for implant-supported complete-arch prosthesis, which were used to determine the search strategy. By using search terms “digital scanning”, “implant”, “complete-arch” and their synonyms, a retrieval formula was established using the logical operators “AND” and “OR” (Table S1). All the data for this study were obtained from a public database, eliminating the need for ethics approval or informed consent.

### 2.3 Bibliometric indicators

In this study, various indicators are used to assess the contribution of countries, journals, authors or the quality of articles such as publication count, annual growth, citation count, co-citation count, impact factor, JCR division, H-index. Visual maps and cluster analysis are used to evaluate the cooperation among countries, journals and authors. The keyword cloud is used to analyze the popularity, novelty and relevance of key topics.

### 2.4 Data analysis and visualization

The data from related articles were obtained and saved in both plain text format and csv (comma separated value) format, comprising information on title, publication year, authors, affiliations, journal of publication, citations, and keywords of these publications. These information were later analyzed and visualized using various tools, including the Online Analysis Platform of Literature Metrology, Scimago Graphica, VOSviewer, and “bibliometrix” package in R software 4.3.1. The Online Analysis Platform visualized publication volume trends over time (https://bibliometric.com/app). Scimago Graphica illustrated collaboration networks among countries/regions [39]. VOSviewer is powerful in visualizing the time trends of countries' cooperation, co-citation references, and keyword frequencies [40]. The “bibliometrix” package was used to analysis the publishing time, publishing journals, authors’ institutions, and keywords. It further visualized the collaborative links among countries, institutions, and authors [41].

## 3 Results

### 3.1 Annual trend in publication growth

During the period from January 1, 1994, to December 24, 2023, a total of 580 publications concerning digital scanning for implant-supported complete-arch prosthesis were identified, including 555 articles and 25 reviews (Fig. S1). The number of publications increased yearly (Fig. 1A), with an annual growth rate of 16.31 %. Related publication outputs remained low before 2018. Beginning in 2018, publication volume in this field surged. Over the last 5 years, annual publications have exceeded 50, highlighting that this field has attracted growing attention. By December 2023, the count reached 80, with expectations for further growth.

**Fig. 1:**
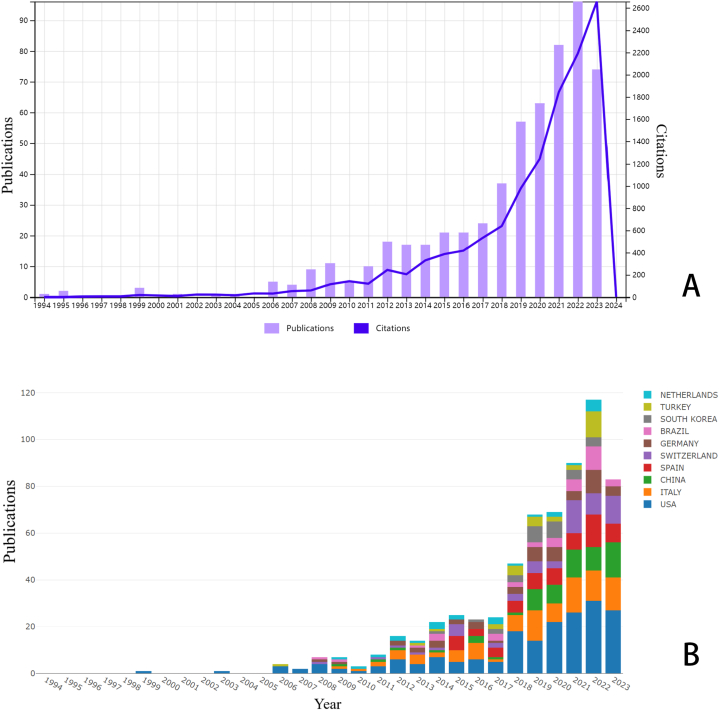
Overview of the related publications in the field of digital scanning for implant-supported complete-arch prosthesis. (A). The annual number of publications from 1994 to 2023 and its growth trend. (B). Trends in the proportion of annual publications in different countries.

### 3.2 Distribution of countries/regions

59 countries and regions were involved in research of digital scanning for implant-supported complete-arch prosthesis. Table 1 and Fig. 1B display the top 10 countries/regions with the highest publication counts. The most productive country/region in this field was the United States (n = 188, 32.41 %), followed by Italy (n = 97, 16.72 %), and Spain (n = 61, 10.52 %). Among the top 10 countries/regions, the United States published the most cited papers with 3910 citations, followed by Italy, which ranked second, with 2353 citations, and Switzerland, which ranked third, with 1199 citations. However, the average number of citations in the Netherlands is 43.58, which is much higher than the other 9 countries, indicating that the articles from the Netherlands appear to be of high quality and influence. It is worth mentioning that China's publication volume has surged in the past five years, and its annual publication volume was second only to the United States and Italy. Although China is actively participating in this field, its average number of citations (10.37) ranked at the bottom among the top 10 countries. In addition, there is exceedingly close international collaboration in this domain. Among the 580 publications, 35.53 % exhibited international collaboration (n = 206), with 57.77 % of these collaborations involving American co-authors (n = 119). The cooperation patterns among various countries/regions, revealing substantial international collaboration, are prominently displayed within European and American nations (Fig. 2, Fig. S2). Notably, collaboration with the US was the most frequent. Node size in Fig. 2 corresponds to each country's publication count, while line thickness indicates the level of cooperation between countries.

**Table 1:** Top 10 productive countries with the highest numbers of publications on digital scanning for implant-supported complete-arch prosthesis.

Rank	Country	Publications (n)	Percentage (n/580)	Citations	Average citations
1	USA	188	32.41 %	3910	20.80
2	Italy	97	16.72 %	2353	24.26
3	Spain	61	10.52 %	940	15.41
4	Switzerland	58	10.00 %	1199	20.67
5	China	51	8.79 %	529	10.37
6	Germany	48	8.28 %	1126	23.46
7	Brazil	35	6.03 %	553	15.80
8	South Korea	29	5.00 %	415	14.31
9	Turkey	28	4.83 %	438	15.64
10	Netherlands	24	4.14 %	1046	43.58

**Fig. 2:**
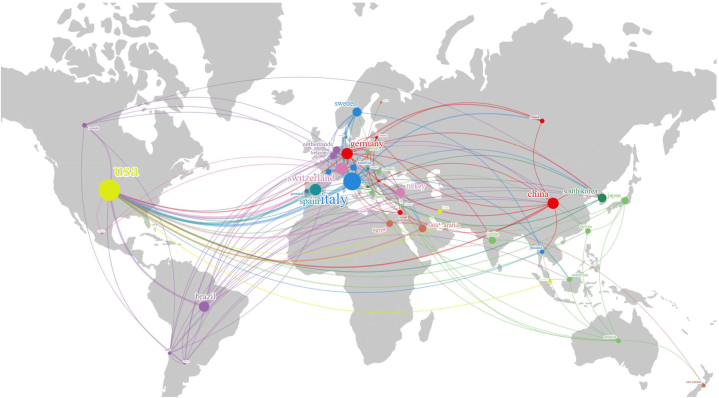
The network map of collaboration relations between countries generated with Scimago Graphica.

### 3.3 Distribution of institutions

**Fig. 3:**
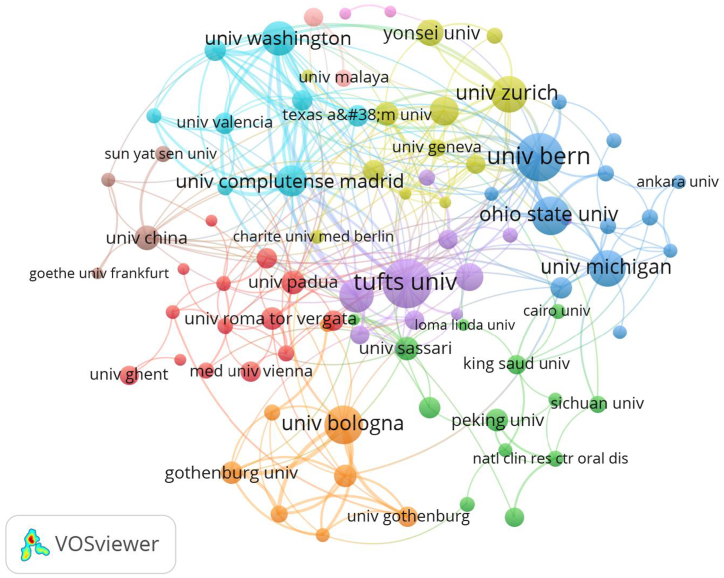
The co-authorship network visualization map of institutions.

Table 2 presents the top 10 institutions with the most publications, half of which are from the United States. According to the number of publications, Tufts University (n = 37) led the top, followed by University of Bern (n = 34), and Ohio State University (n = 30). However, when considering average citations, the top three shifted to University of Michigan (33.67), Tufts University (32.16), and University of Zurich (29.38). VOSviewer was utilized for analyzing and visualizing the institutions driving advancements in the field. Fig. 3 visualized the co-authorship network map of institutions, where distinct colors signify various clusters. Cooperation among major institutions showed a regional tendency. For instance, the institutions in Chinese-speaking areas tended to collaborate more within those Chinese -speaking countries.

**Table 2:** Top 10 institutions with the most publications of digital scanning for implant-supported complete-arch prosthesis.

Rank	Institution	Country	Publications	Citations	Average citations
1	Tufts University	USA	37	1190	32.16
2	University of Bern	Switzerland	34	533	15.68
3	Ohio State University	USA	30	438	14.60
4	University of Washington	USA	25	292	11.68
5	University of Rochester	USA	25	378	15.12
6	Complutense University of Madrid	Spain	21	392	18.67
7	University of Zurich	Switzerland	21	617	29.38
8	University of Michigan	USA	15	505	33.67
9	University of Padua	Italy	13	226	17.38
10	Yonsei University	South Korea	13	170	13.08

### 3.4 High yield and highly-cited journals

**Fig. 4:**
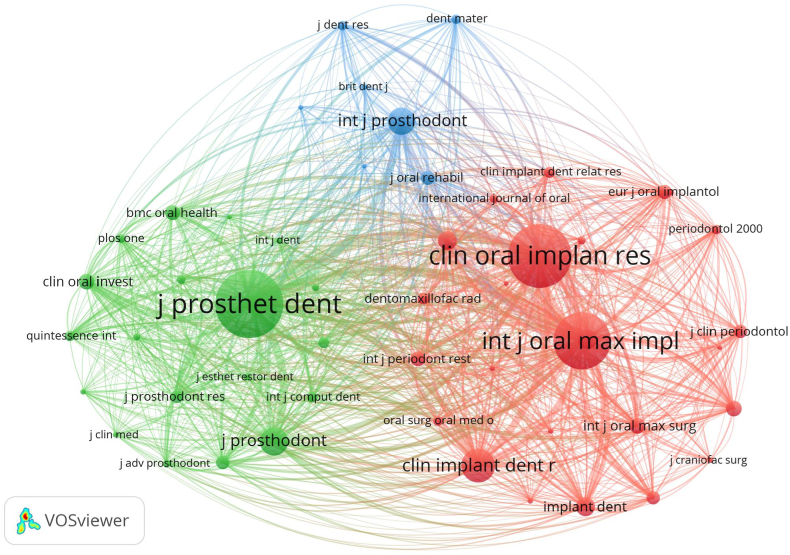
The co-authorship map of high-yielding journals.

During the period from January 01, 1994 to December 24, 2023, 580 publications on research concerning digital scanning for implant-supported complete-arch prosthesis appeared in 70 journals, with 24 journals featuring at least 5 publications. An analysis of co-authorship in the journals was carried out, and the results are presented in the network map in Fig. 4. Among the 580 publications, the Journal of Prosthetic Dentistry (4.6, Q1) published the highest number (Table 3), with 105 articles (18.10 % of the total), followed by Journal of Prosthodontics-Implant Esthetic and Reconstructive Dentistry (4.0, Q1), and Clinical Oral Implants Research (4.3, Q1). Moreover, by leveraging VOSviewer data and the Journal Citation Reports (JCR) evaluation system, we observed that the top 10 journals primarily belong to the Q1 category, suggesting that the majority of the publications were published in prestigious and impactful journals. Regarding citations, the top three changed to Journal of Prosthetic Dentistry (4.6, Q1), Clinical Oral Implants Research (4.3, Q1), and International Journal of Oral & Maxillofacial Implants (2.0, Q3) (Table 3). Besides, 4 of the top 10 journals had an IF above 4.

**Table 3:** Top 10 high-yield and high-cited journals related to digital scanning for implant-supported complete-arch prosthesis.

Rank	Journal	IF and JCR division (2022)	Publications	Citations	Average citations
**High-yield journals**					
1	Journal of Prosthetic Dentistry	4.6, Q1	105	2186	20.82
2	Journal of Prosthodontics-Implant Esthetic and Reconstructive Dentistry	4.0, Q1	58	1183	20.40
3	Clinical Oral Implants Research	4.3, Q1	51	1952	38.27
4	International Journal of Oral & Maxillofacial Implants	2.0, Q3	44	1608	36.55
5	Journal of Dentistry	4.4, Q1	32	229	7.16
6	Clinical Implant Dentistry and Related Research	3.6, Q1	24	652	27.17
7	International Journal of Prosthodontics	2.3, Q3	15	331	22.07
8	Journal of Clinical Medicine	3.9, Q2	15	117	7.80
9	Journal of Esthetic and Restorative Dentistry	3.2, Q2	15	193	12.87
10	Bmc Oral Health	2.9, Q2	14	527	37.64
**High-cited journals**					
1	Journal of Prosthetic Dentistry	4.6, Q1	105	2186	20.82
2	Clinical Oral Implants Research	4.3, Q1	51	1952	38.27
3	International Journal of Oral & Maxillofacial Implants	2.0, Q3	44	1608	36.55
4	Journal of Prosthodontics-Implant Esthetic and Reconstructive Dentistry	4.0, Q1	58	1183	20.40
5	Clinical Implant Dentistry and Related Research	3.6, Q1	24	652	27.17
6	Bmc Oral Health	2.9, Q2	14	527	37.64
7	Plos One	3.7, Q2	4	345	86.25
8	International Journal of Prosthodontics	2.3, Q3	15	331	22.07
9	Journal of Prosthodontic Research	3.6, Q1	10	304	30.40
10	Journal of Dentistry	4.4, Q1	32	229	7.16

### 3.5 High impact researchers

**Fig. 5:**
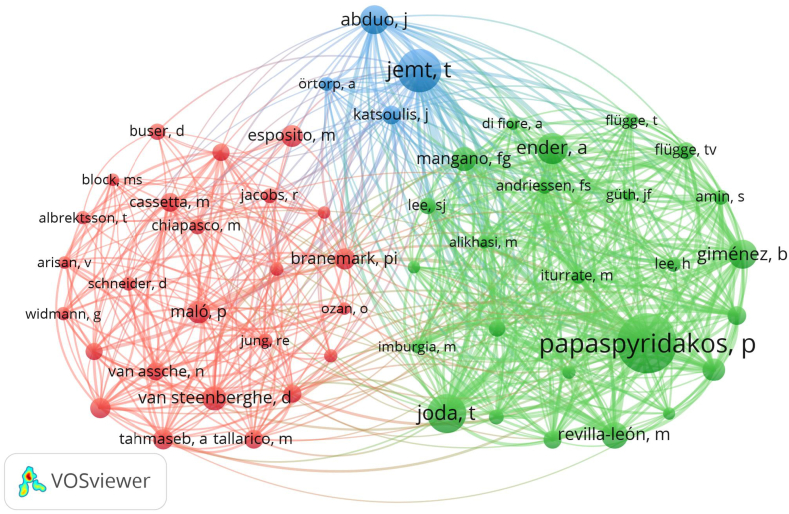
The network map of collaboration relations between authors.

A total of 1896 researchers contributed to studies on digital scanning for implant-supported complete-arch prosthesis. The top 10 productive authors are listed in Table 4. Papaspyridakos P was the most productive researcher with 31 publications and 1213 citations. And there is a clear gap between Papaspyridakos P and other authors from the perspective of H-index and citation. His research team had the highest citation count, with their articles cited 1213 times, averaging 39.13 citations each, which indicated that Papaspyridakos P's team had made great contributions to this field. Notably, top 10 authors were almost all from USA. In addition, as shown in Fig. 5, there is a rich global level of collaborative relationships among the authors.

**Table 4:** Top 10 authors with the most publications on digital scanning for implant-supported complete-arch prosthesis.

Rank	Author	H-index	Publications	Citations	Average citations
1	Papaspyridakos P	19	31	1213	39.13
2	Yilmaz B	9	25	307	12.28
3	Revilla-leon M	9	21	225	10.71
4	Pozzi A	10	14	375	26.79
5	Chochlidakis K	7	13	220	16.92
6	Wang HL	8	12	140	11.67
7	Weber HP	10	11	421	38.27
8	Gomez-polo M	5	11	115	10.45
9	Agustin-panadero R	6	11	117	10.64
10	Finkelman M	7	10	324	32.40

### 3.6 Highly-cited papers and keywords trends

The information is presented in Table 5 regarding the 10 most frequently co-cited references. Co-cited references denote references that are cited by multiple retrieved articles, suggesting a certain degree of thematic similarity or relevance among the respective articles [42]. The most co-cited reference was “Accuracy of digital impressions of multiple dental implants: an in vitro study”, produced by Vandeweghe S, published in Clinical Oral Implants Research in 2017 (79 times). Vandeweghe S assessed the accuracy of 4 intraoral scanners for implant scans in the edentulous jaw and found that not all scanners are appropriate for capturing implant scans for a full-arch bridge in the edentulous jaw. Similarly, the remaining 9 references are also concerning about the accuracy analysis of intraoral scanning for edentulous jaw. This search result fully shows that accuracy is a hotspot of high interest in this field.

**Table 5:** The top 10 most co-cited publications associated with digital scanning for implant-supported complete-arch prosthesis from 1994 to 2023.

Rank	Cited Reference	Author	Year	Journal	IF (2022)	DOI	Co-citations
1	Accuracy of digital impressions of multiple dental implants: an in vitro study	Vandeweghe S	2017	Clinical Oral Implants Research	4.3	10.1111/clr.12853	79
2	Digital versus conventional implant impressions for edentulous patients: accuracy outcomes	Papaspyridakos P	2016	Clinical Oral Implants Research	4.3	10.1111/clr.12567	75
3	Applicability and accuracy of an intraoral scanner for scanning multiple implants in edentulous mandibles: a pilot study	Andriessen FS	2014	Journal of Prosthetic Dentistry	4.6	10.1016/j.prosdent.2013.07.010	70
4	Digital vs. conventional full-arch implant impressions: a comparative study	Amin S	2017	Clinical Oral Implants Research	4.3	10.1111/clr.12994	65
5	Intraoral scan bodies in implant dentistry: A systematic review	Mizumoto RM	2018	Journal of Prosthetic Dentistry	4.6	10.1016/j.prosdent.2017.10.029	56
6	The accuracy of implant impressions: a systematic review	Lee H	2008	Journal of Prosthetic Dentistry	4.6	10.1016/s0022-3913(08)60208-5	56
7	Accuracy of four intraoral scanners in oral implantology: a comparative in vitro study	Imburgia M	2017	Bmc Oral Health	2.9	10.1186/s12903-017-0383-4	56
8	Accuracy of a digital impression system based on parallel confocal laser technology for implants with consideration of operator experience and implant angulation and depth	Giménez B	2014	International Journal of Oral & Maxillofacial Implants	2.0	10.11607/jomi.3343	55
9	Accuracy of a digital impression system based on active wavefront sampling technology for implants considering operator experience, implant angulation, and depth	Giménez B	2015	Clinical Implant Dentistry and Related Research	3.6	10.1111/cid.12124	52
10	Digital Versus Conventional Impressions in Fixed Prosthodontics: A Review	Ahlholm P	2018	Journal of prosthodontics	4.0	10.1111/jopr.12527	51

**Fig. 6:**
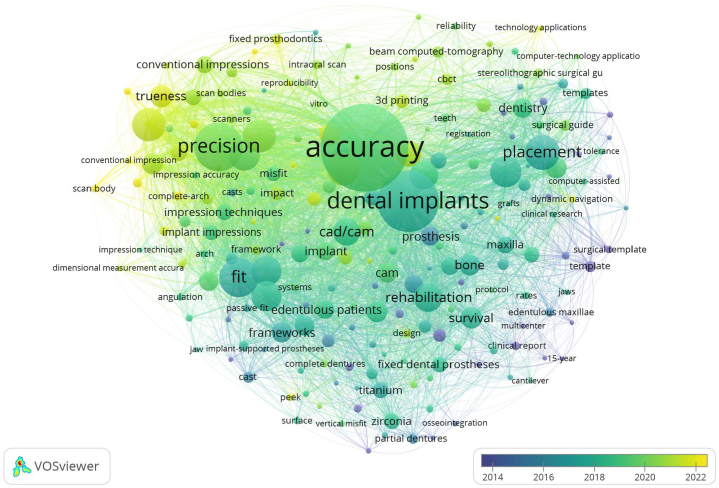
The keyword cloud of the publications.

Keyword analysis also yielded similar results, which can also display research hotspots and directions. The three most common keywords were “accuracy” (228 occurrences), “dental implants” (140 occurrences) and “precision” (103 occurrences). Through further visualizing text analysis results with keyword clouds, we also found that “accuracy” was the core word in this field. The temporal distribution of keywords was further illustrated to highlight changes in research hotspots over time (Fig. 6). The latest keywords included “intraoral scanners”, “trueness”, “scan body”, “photogrammetry”, “strategies” and so on.

## 4 Discussion

### 4.1 General information

The conventional impression is considered to be the standard care clinically for edentulous arch based on its high accuracy, but complex procedures are needed. Digital scanning based on intraoral scanning and photogrammetry might provide a clinically acceptable alternative to conventional complete-arch implant impression. The increasing number of annual publications indicates that it is a hot topic in prosthetic dentistry.

Digital scans are typically obtained through IOS, which has been widely used for single implant restorations [10,43]. However, challenges arose when it's used for edentulous arches, attributed to the lack of anatomic indexes, reflectivity and inherent mobility of soft tissue, similar shape of the scan bodies, and the long scanning path [22,24,[44], [45], [46], [47], [48], [49], [50], [51], [52], [53], [54], [55], [56]]. The 2018 ITI Consensus Report stated that it is still not recommended to routinely use intraoral digital implant scans clinically to large interimplant spans or edentulous arches due to insufficient accuracy [10]. The annual number of publications before 2018 (less than 25) also indicates that IOS has not yet been widely adopted in this field at that time.

The annual number of publications has increased rapidly since 2018, which can be attributed to two reasons. On the one hand, many researchers have proposed methods to improve the accuracy of intraoral digital scans for edentulous arches. The common strategy of these methods is to add easily identifiable connections between dispersed scan bodies with characteristic structures or modify the shape of the scan bodies, which has improved the accuracy of intraoral digital scans for edentulous arches and surpassed conventional open-tray splinted impressions in some cases [14,25,47,[57], [58], [59], [60], [61], [62], [63], [64], [65], [66], [67], [68], [69], [70], [71], [72]]. On the other hand, photogrammetry has garnered particular attention in this field recently, providing a digital alternative to accurately recording the 3D positions of dental implants [[73], [74], [75], [76], [77], [78], [79], [80]]. Photogrammetry is a technique that uses the device equipped with multiple cameras to capture the target object from multiple angles, thereby acquiring the 3D positions of the target object [81]. It has demonstrated high accuracy in other disciplines such as topographic surveying, automobile manufacturing, and civil engineering previously [82,83]. Subsequently, this technique has been used for acquiring 3D implant positions for partial or completely edentulous arches [34,82,[84], [85], [86], [87], [88], [89], [90], [91], [92], [93], [94]]. Due to its operational mechanism that eliminates the necessity for overlapping captured images, it may not be influenced by factors such as scanning patterns and ambient illumination conditions, easing the digitizing method [87,91,94,95].

As a commonly used bibliometric indicator, the citation count is a numerical parameter to quantify the impact of a publication. The co-citation count represents the popularity of a reference by researchers in the same field, indicating the quality and impact of the research, offering clinicians an objective basis for making evidence-based decisions. Based on this criterion and other indicators, we believe that Papaspyridakos P is the most contributing author and Journal of Prosthetic Dentistry is the scientific journal with the highest impact in this field. However, it should be noted that citation count is a continuously growing parameter that is influenced by publication time, so we may underestimate the quality of some newer articles. For example, Revilla-Leon M has published many articles in this field and has a high H-index, but has received relatively few citations. This may be because his articles are mainly published in 2016–2023, which are relatively new. The top 10 most co-cited references focused on clinical applications of IOS. This may be attributed to the relatively early application of IOS, leading to a greater volume of publications and earlier publication dates. In contrast, publications on the relatively new stereophotogrammetry technique are still limited and have not yet received significant citations.

### 4.2 Hotspots and frontiers

The keywords analysis is essential for identifying research interests and trends. In this field, “accuracy” is the core word, which is crucial for the long-term success of implant-supported complete-arch prosthesis, as insufficient accuracy may lead to a misfit of the prosthesis, ultimately resulting in potential mechanical and biological complications [[3], [4], [5], [6]]. Other keywords related to accuracy include “precision”, “fit”, “misfit” and “placement”, while those related to prognosis include “bone”, “survival”, and “follow-up”. To assess accuracy, researchers often compare the implant positions acquired from photogrammetry with those from the 3D models acquired by IOS or conventional impression scanned by a laboratory scanner [96]. Relevant 8 in vitro and in vivo studies have been summarized in Table 6 [2,34,95,[97], [98], [99], [100], [101]]. Most of the above results consistently indicated that both the trueness and precision of photogrammetry were superior to those of IOS or conventional impression [34,[97], [98], [99], [100], [101]]. However, Revilla-León came to the opposite conclusion, that photogrammetry is the least accurate of the three techniques [2]. The comparison results of IOS and conventional impression are also inconsistent. The inconsistencies of the above conclusions may arise from differences in scanner types, accuracy evaluation parameters and software prograte the impact of different systems, am or algorithms [34]. Due to the heterogeneity of different experimental designs, further research is still needed to evaluaccuracy evaluation methods, number and angle of implants, connection of accessories and other factors on the accuracy of implant positions acquired by photogrammetry.

**Table 6:** Characteristics of in vitro and in vivo studies comparing conventional, intraoral scanning, and photogrammetry accuracy of complete-arch implant impression.

Reference	Study type	Groups and systems	Evaluation parameters	Accuracy	Conclusions
(Revilla-León et al., 2021)	In vitro (n = 10)	PG: iCam4D; Imetric. IOS: iTero Element; Cadent. TRIOS 3; 3Shape A/S. CI: Splinting AM metal framework, AM custom tray and polyether impression technique. Reference coordinates: CMM; Contura G2; Zeiss. CAD software program: Geomagic; 3D Systems.	Linear deviations, angular deviations and 3D deviations between the final cast and scanned models.	Linear deviations (x-axis, y-axis, z-axis): (p = 0.004) PG (23.8 μm, 73.7 μm, −4.7 μm) CI (7.3 μm, 8.9 μm, 1.8 μm) IOS-iTero Element (4.1 μm, 17.5 μm, −4.1 μm) IOS-TRIOS 3 (9.7 μm, 18.0 μm, −4.9 μm) Angular deviations (XZ angle, YZ angle): (p < 0.001) PG (0.1°, 0.3°) CI (0.1°, −0.1°) IOS-iTero Element (−0.1°, −0.1°) IOS-TRIOS 3 (0.2°, 0.2°) 3D deviations: PG: 77.6 μm; CI: 11.7 μm; IOS-iTero Element: 18.4 μm; IOS-TRIOS 3: 21.1 μm.	PG showed the worst accuracy outcome with the highest 3D discrepancy for the implant abutment positions of all the groups. The CI group obtained the best accuracy outcome among all the groups.
(Ma et al., 2021)	In vitro (n = 10)	PG: iCam4D; Imetric. IOS: TRIOS3; 3Shape A/S. CI: Splinted open-tray impression digitized by E4; 3Shape A/S. CAD software program: Geomagic Control X; 3D Systems.	3D deviations of implant replica positions.	3D deviations (Trueness, Precision): (p < 0.001) PG (24.45 μm, 2.00 μm) CI (28.70 μm, 29.40 μm) IOS (43.45 μm, 36.00 μm)	For complete-arch implant rehabilitation, PG showed the highest accuracy of all the groups, followed by CI, and IOS provided the least accuracy.
(Zhang et al., 2021)	In vivo (n = 14)	PG: iCam4D; Imetric. CI: Splinted open-tray impression. (Digitized by laboratory scanner (LS3; Nobel Biocare)) CAD software program: Geomagic Studio 2014; 3D System.	Distance deviation and angular deviation between PG and CI models.	PG Trueness: Linear deviations (mean ± SD): 70 ± 57 μm. Angular deviations (mean ± SD): 0.432 ± 0.348°.	PG deviation trueness within the clinically acceptable error range of 150 μm.
(Sallorenzo et al., 2022)	In vitro (n = 10)	PG: PIC system; PICdental. IOS: TRIOS3; 3Shape A/S. Reference coordinates: CMM. CAD software program: (Geomagic; 3D Systems).	Linear deviations and angular deviations of parallel or angled implant positions.	Linear deviations (Trueness, Precision) Parallel implant cast: PG (20 μm, 32 μm) IOS (100 μm, 292 μm) Angled implant cast: PG (10 μm, 65 μm) IOS (23 μm, 205 μm) Angular deviations (Trueness, Precision) Parallel implant cast: PG (0.354°, 0.280°) IOS (1.177°, 0.474°) Angled implant cast: PG (0.084°, 0.246°) IOS (0.529°, 0.841°)	PG showed higher precision and trueness than IOS for scanning complete-arch implant-supported prostheses.
(Kosago et al., 2022)	In vitro (n = 5)	PG:PIC System; PIC Dental. IOS-1: TRIOS 4; 3Shape. IOS-2: iTero Element 2; Align Technology. IOS-3: Primescan; Dentsply-Sirona. CI: Open-tray splint impression with single-mixed single impression digitized by E4; 3Shape A/S. CAD software program: Geomagic Control X; 3D Systems.	3D deviations of scan body positions.	3D deviations (Trueness, Precision): (P < 0.05) PG (48.74 μm, 5.46 μm) IOS-1 (52.14 μm, 19.39 μm) IOS-2 (67.72 μm, 36.84 μm) IOS-3 (57.24 μm, 28.85 μm) CI (141.86 μm, 49.40 μm).	For completed-arch digital implant impressions, PG presents higher accuracy than other techniques, especially in terms of precision. CI showed the lowest trueness and precision.
(Orejas et al., 2022)	In vivo (n = 5)	PG: PIC System; PIC Dental. IOS-1: TRIOS 3; 3Shape A/S. IOS-2: True Definition; 3M ESPE. CAD software program: Geomagic; 3D Systems.	Linear deviations and angular deviations of implant positions.	Precision (Maxilla/Mandible): Linear: PG (108.2/104.4) μm, IOS-1 (220.6/476.4) μm, IOS-2 (342.9/269.0) μm. Angular: PG (0.44/0.24) degrees, IOS-1 (1.26/2.57) degrees, IOS-2 (1.01/1.61) degrees.	PG system had better precision than the IOS systems.
(Revilla-León et al., 2023)	In vitro (n = 10)	PG: PIC System; PIC Dental. CI: Splinting AM metal framework, AM custom tray and elastomeric impression technique. Reference coordinates: CMM; Contura G2; Zeiss. CAD software program: Calypso; Carl Zeiss.	Linear deviations (x-, y-, and z-axes), angular deviations (XZ and YZ angle) and 3D deviations between positions of implant abutments with reference cast.	Linear deviations (x-axis, y-axis, z-axis): (p < 0.001) PG (13.36 μm, 11.47 μm, 8.05 μm) CI (11.18 μm, 11.86 μm, 2.82 μm) Angular deviations (XZ angle, YZ angle): (p < 0.001) PG (0.03°, 0.08°) CI (0.22°, 0.26°) 3D deviations: PG: 20.15 μm; CI: 18.40 μm.	CI showed higher accuracy than PG.
(Tohme et al., 2023)	In vitro (n = 15)	PG: PIC System; PIC Dental.IOS: TRIOS3; 3Shape A/S.CI: Abutment-level impression digitized by E3; 3Shape A/S. CAD: Geomagic Control X; 3D System.	Angular deviation of whole scan bodies. Whole scan bodies and flat angled surface' 3D deviations.	Angular deviation (Trueness, Precision): (P < 0.001) PG (0.809°, 0.010°) CI (0.922°, 1.142°) IOS (1.081°, 0.221°) 3D deviation (Trueness, Precision): (P < 0.001) whole scan bodies: PG (88 μm, 2 μm) CI (115 μm, 103 μm) IOS (148 μm, 63 μm) flat angled surface: PG (213 μm, 2 μm) CI (166, 166 μm) IOS (112 μm, 67 μm)	For whole scan bodies 3D deviation and angular deviation, PG showed highest trueness and precision. But for the flat angled surface, IOS showed higher trueness.

PG: Photogrammetry; CI: Conventional impression; IOS: Intraoral scanning; CMM: Coordinate measuring machine; CAD, computer-aided design; IQR, interquartile range.

In addition, high equipment costs and the level of technological development may be limiting factors for the widespread application of digital scanning, which explains why high-income countries have larger volumes of publications with higher average citation counts. This is due to the fact that after acquiring digital scans, further processes such as data preprocessing, multi-source data integration, digital design, and final prosthesis manufacturing are required. This process involves a variety of expensive digital equipment, advanced software programs, and well-trained operators, where developed countries have an advantage [48]. The analysis outcomes indicate that digital scanning technology is more popular in developed countries, with the United States in a leading position. Judging from the international cooperation network, the United States is also the country that cooperates most frequently with other countries, which is very helpful for data exchange and the development of relevant techniques. Therefore, we should be aware that the development of economy and the upgrading of digital equipment will accelerate the transformation of conventional impressions to digital scans for implant-supported complete-arch prosthesis, leading to changes in related research topics.

### 4.3 Limitations of this study

This study is the initial extensive bibliometric analysis of the digital scanning technique for implant-supported complete-arch prosthesis. However, there are notable limitations to acknowledge. Firstly, the search confined to the WoSCC database and the restriction to English-language publications may have omitted some studies. The exclusive reliance on published sources potentially leads to publication bias. Secondly, the public database was not specifically designed for bibliometric analysis, thus the errors it may contain will affect the analysis performed on downloaded data [102]. Thirdly, while bibliometric analysis is inherently quantitative, qualitative statements from bibliometrics still exhibit considerable subjectivity [102]. Additionally, bibliometric analysis can only assess the short-term development trends of a research field, but it cannot provide a long-term prediction.

## 5 Conclusions

Digital scanning for implant-supported complete-arch prosthesis is a hot topic in prosthetic dentistry. The United States plays a leading role in this field, which is closely related to its high economic level, technological level and close international cooperation. Papaspyridakos P is the most contributing author and Journal of Prosthetic Dentistry is the scientific journal with the highest impact in this field. Accuracy was the core word in this field, and it is likely to continue to be a research hotspot in the future. More scientific evidence is still needed to support the clinical application of digital scanning in this field. Moreover, collaboration and communication between scientists, countries, and organizations must improve to promote progress in these research areas and identify new hotspots for exploration.

## Ethics approval statement

Not applicable. Wuhan University does not require approval for bibliometric studies.

## Data availability statement

The original contributions presented in the study are included in the article, and further inquiries can be directed to the corresponding author.

## Funding

This research was supported by Wuhan University Graduate Tutor Education Method Innovation Project.

## Consent for publication

Not applicable.

## CRediT authorship contribution statement

**Yutong Gao:** Writing – original draft, Validation, Software, Project administration, Data curation, Conceptualization. **Mingyu Zhao:** Writing – original draft, Visualization, Methodology, Formal analysis. **Shici Xia:** Supervision, Methodology, Investigation, Conceptualization. **Yue Sa:** Writing – review & editing, Resources, Project administration, Methodology, Conceptualization.

## Declaration of competing interest

The authors declare that they have no known competing financial interests or personal relationships that could have appeared to influence the work reported in this paper.

## Figures and Tables

**figs1:**
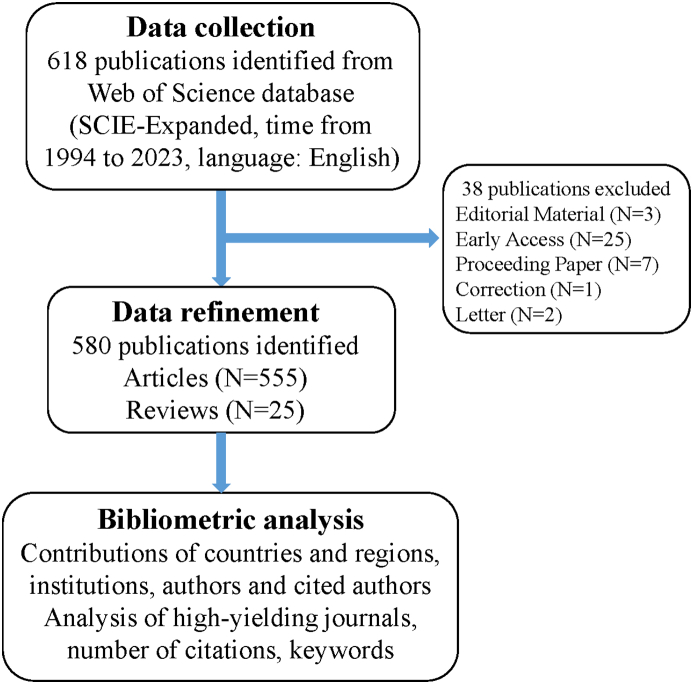


**figs2:**
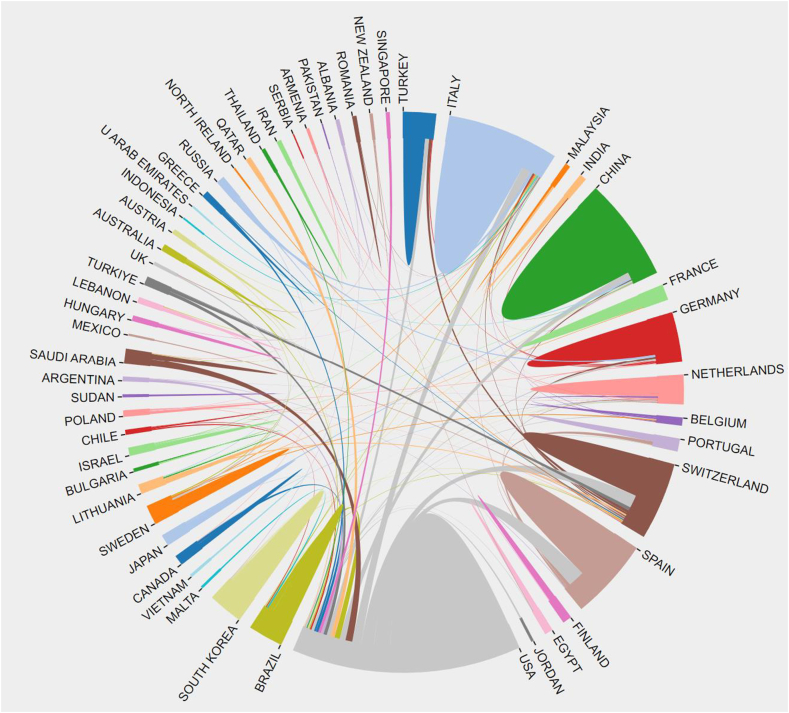


## Abbreviations

IOS: Intraoral scanning
WoSCC: Web of Science Core Collection
SCI-E: Citation Index Expanded
JCR: Journal citation reports
