# Supplementary material for: Knowledge structure and research hotspots on digital scanning for implant-supported complete-arch prosthesis: A bibliometric analysis
Source: Heliyon. 2024 Aug 23;10(17):e36782. doi: 10.1016/j.heliyon.2024.e36782 (PMC11402722; doi:10.1016/j.heliyon.2024.e36782)
Supplement: Multimedia component 1 [file mmc1.docx]

**Supplementary Table 1. The search strategy of Web of Science Core Collection (WoSCC).**

| Research database | Web of Science Core Collection (WoSCC) |
| --- | --- |
| Citation indexes | Science Citation Index Expanded (SCI-EXPANDED) |
| Query formulation | ((TI=(implant*)) OR (AB=(implant*)) OR (AK=(implant*))) AND ((TI=((digital-impression*) OR (optical-impression*) OR (digital-implant-impression*) OR (dental-impression-technique*) OR (dental-model*) OR (digital-scan*) OR (dental-scan*) OR (intraoral-scan*) OR (extraoral-scan*)OR (continuous-scan*) OR (optical-scan*) OR (scan-bod*) OR (scanner*) OR (IOS) OR (digitizer) OR (computer-aided-design*) OR (CAD/CAM) OR (photogrammetr*) OR (stereophotogrammetr*) OR (digital-dentistry) OR (digital-workflow))) OR (AB=((digital-impression*) OR (optical-impression*) OR (digital-implant-impression*) OR (dental-impression-technique*) OR (dental-model*) OR (digital-scan*) OR (dental-scan*) OR (intraoral-scan*) OR (extraoral-scan*)OR (continuous-scan*) OR (optical-scan*) OR (scan-bod*) OR (scanner*) OR (IOS) OR (digitizer) OR (computer-aided-design*) OR (CAD/CAM) OR (photogrammetr*) OR (stereophotogrammetr*) OR (digital-dentistry) OR (digital-workflow))) OR (AK=((digital-impression*) OR (optical-impression*) OR (digital-implant-impression*) OR (dental-impression-technique*) OR (dental-model*) OR (digital-scan*) OR (dental-scan*) OR (intraoral-scan*) OR (extraoral-scan*)OR (continuous-scan*) OR (optical-scan*) OR (scan-bod*) OR (scanner*) OR (IOS) OR (digitizer) OR (computer-aided-design*) OR (CAD/CAM) OR (photogrammetr*) OR (stereophotogrammetr*) OR (digital-dentistry) OR (digital-workflow)))) AND ((TI=((edentulous) OR (full-arch*) OR (fullarch*) OR (complete-arch*) OR (all-on-4) OR (all-on-four) OR (all-on-6) OR (all-on-six))) OR (AB=((edentulous) OR (full-arch*) OR (fullarch*) OR (complete-arch*) OR (all-on-4) OR (all-on-four) OR (all-on-6) OR (all-on-six))) OR (AK=((edentulous) OR (full-arch*) OR (fullarch*) OR (complete-arch*) OR (all-on-4) OR (all-on-four) OR (all-on-6) OR (all-on-six)))) |
| Language | English |
| Document types | Articles and Reviews |
| Searching period | January 1, 1994 to December 24, 2023 |
| Data collection | Export with full records and cite reference in plain text format |
| Sample size | 580 publications including 555 articles and 25 reviews |

*Wildcard
